# Mechanistic insights into ectodomain shedding: susceptibility of CADM1 adhesion molecule is determined by alternative splicing and *O*-glycosylation

**DOI:** 10.1038/srep46174

**Published:** 2017-04-10

**Authors:** Kyoko Shirakabe, Takuya Omura, Yoshio Shibagaki, Emiko Mihara, Keiichi Homma, Yukinari Kato, Akihiko Yoshimura, Yoshinori Murakami, Junichi Takagi, Seisuke Hattori, Yoshihiro Ogawa

**Affiliations:** 1 Department of Organ Network and Metabolism, Graduate School of Medical and Dental Sciences, Tokyo Medical and Dental University (TMDU), Tokyo 113-8510, Japan; 2 Department of Molecular Endocrinology and Metabolism, Graduate School of Medical and Dental Sciences, Tokyo Medical and Dental University (TMDU), Tokyo 113-8510, Japan; 3 Division of Biochemistry, School of Pharmaceutical Science, Kitasato University, Tokyo 108-8641, Japan; 4 Laboratory of Protein Synthesis and Expression, Institute for Protein Research, Osaka University, Osaka 565-0871, Japan; 5 Department of Life Science and Informatics, Maebashi Institute of Technology, Maebashi 371-0816, Japan; 6 Department of Regional Innovation, Tohoku University Graduate School of Medicine, Sendai 980-8575, Japan; 7 Department of Microbiology and Immunology, Keio University School of Medicine, Tokyo 160-8582, Japan; 8 Division of Molecular Pathology, Institute of Medical Science, The University of Tokyo, Tokyo 153-8902, Japan; 9 Department of Medical and Bioregulatory Science, Graduate School of Medical Sciences, Kyushu University, Fukuoka 812-8582, Japan; 10 Japan Agency for Medical Research and Development, CREST, Tokyo 100-0004, Japan

## Abstract

Ectodomain shedding (shedding) is a post-translational modification, which liberates the extracellular domain of membrane proteins through juxtamembrane processing executed mainly by the ADAM (a disintegrin and metalloprotease) family of metalloproteases. Because shedding alters characteristics of cells in a rapid and irreversible manner, it should be strictly regulated. However, the molecular mechanisms determining membrane protein susceptibility to shedding (shedding susceptibility) are largely unknown. Here we report that alternative splicing can give rise to both shedding-susceptible and shedding-resistant CADM1 (cell adhesion molecule 1) variant proteins. We further show that *O*-glycans adjacent to the shedding cleavage site interfere with CADM1 shedding, and the only 33-bp alternative exon confers shedding susceptibility to CADM1 by inserting five non-glycosylatable amino acids between interfering *O*-glycans and the shedding cleavage site. These results demonstrate that shedding susceptibility of membrane protein can be determined at two different levels of its biosynthesis pathway, alternative splicing and *O*-glycosylation.

Ectodomain shedding, also simply called shedding, is a post-translational modification mechanism, which liberates the extracellular domain of membrane proteins through juxtamembrane processing executed mainly by ADAMs (a disintegrin and metalloproteases), a family of membrane-anchored metalloproteases^1^,^2^,^3^,^4^. Shedding can release growth factors and cytokines that are expressed as the extracellular domain of membrane proteins, thereby drastically expanding their effective working area. On the other hand, shedding can decrease the amount of cell surface receptors and adhesion molecules with a concomitant release of soluble decoy proteins, thereby reducing the responsiveness of cells to their cognate ligands. In addition, membrane-remaining shedding products are often subjected to γ-secretase-dependent intramembrane proteolysis^5^, which generates cleaved intracellular domains that translocate to the nucleus and thus regulate gene transcription. Taken together, shedding is a unique post-translational modification mechanism, which can induce a variety of cellular responses through a single processing event.

Since shedding is such an effective and immediate mechanism, it should be strictly regulated. In addition, since shedding is an adaptive response to environmental conditions, physiologically relevant membrane proteins (e.g. a pro-inflammatory cytokine TNFα) should be selectively shed in response to extracellular stimuli (e.g. bacterial endotoxin lipopolysaccharide (LPS))^4^. However, the molecular mechanisms that determine susceptibility to shedding of membrane proteins (shedding susceptibility) are largely unknown. It is noteworthy that shedding target membrane proteins reported to date have no consensus cleavage site sequences. Accordingly, two major sheddases, ADAM10 and ADAM17, only have broad preference to amino acid types and require no specific amino acids at any positions surrounding the cleavage site^6^. On the other hand, extensive mutations of the cleavage site can prevent shedding of some membrane proteins^7^,^8^,^9^, thus it is supposed that scissile bond sequence is required for shedding susceptibility of membrane proteins. Meanwhile, the stalk length, which is defined as the length between the cell surface and the membrane-proximal extracellular domain of membrane proteins, is also considered to be a determinant of shedding susceptibility, since membrane proteins having shorter stalk region are mostly resistant to shedding^8^,^10^,^11^. It is, therefore, likely that both scissile bond sequence and sufficient stalk length are required for shedding susceptibility of membrane proteins, however these factors cannot completely explain the high selectivity of naturally occurring shedding.

To further elucidate the molecular mechanisms determining the shedding susceptibility of membrane proteins, comprehensive analysis of naturally occurring shedding is indispensable. Thus we previously developed a proteomic screening system for shedding target proteins using 2D-DIGE (two-dimensional difference gel electrophoresis) and reported that multiple membrane proteins are simultaneously shed in the LPS-stimulated macrophage cell line^12^,^13^. In this study, we employ another quantitative method called SILAC (stable isotope labeling with amino acids in cell culture) and identify additional membrane proteins naturally shed in LPS-stimulated macrophages. In addition, through the detailed analysis of a newly identified shedding target, CADM1 (cell adhesion molecule 1), we find that the shedding susceptibility of CADM1 depends entirely on the inclusion of the only 33-bp alternative exon, exon 9. We further show that *O*-glycans adjacent to the shedding cleavage site interfere with shedding of CADM1 and that exon 9 makes CADM1 susceptible to shedding by inserting five non-glycosylatable amino acids between interfering *O*-glycans and the shedding cleavage site. This study provides evidence that shedding susceptibility of membrane protein is regulated at two different levels of its biosynthesis pathway, post-transcriptional alternative splicing and post-translational *O*-glycosylation.

## Results

### Proteomic screening of shedding targets in LPS-stimulated macrophages using SILAC

We employed a stable isotope-based quantitative proteomic method called SILAC^14^ to screen shedding target proteins which are released from LPS-stimulated murine macrophage Raw 264.7 cells in a metalloprotease-dependent manner using a broad metalloprotease inhibitor, BB94. *N*-glycosylated peptides derived from conditioned media obtained from LPS− (heavy, H) or LPS + BB94− (light, L) treated cells were quantified and compared using LC-MS/MS (Supplementary Fig. 1). A total of 557 quantified *N*-glycosylated peptides were assigned to 87 unique proteins, and 18 proteins (20.7%) with the geometric mean H/L ratio >1.5 were defined as candidate shedding targets (Table 1). They included four shedding targets identified in our previous screening using 2D-DIGE^12^,^13^, indicating the efficacy and reliability of the screening system. All the proteins had transmembrane domain(s), 14 of which (77.8%) contained immunoglobulin (Ig)-like domain(s) in their extracellular domains.

### Alternative splicing can give rise to both shedding-susceptible and shedding-resistant CADM1 variant proteins

**Figure 1:**
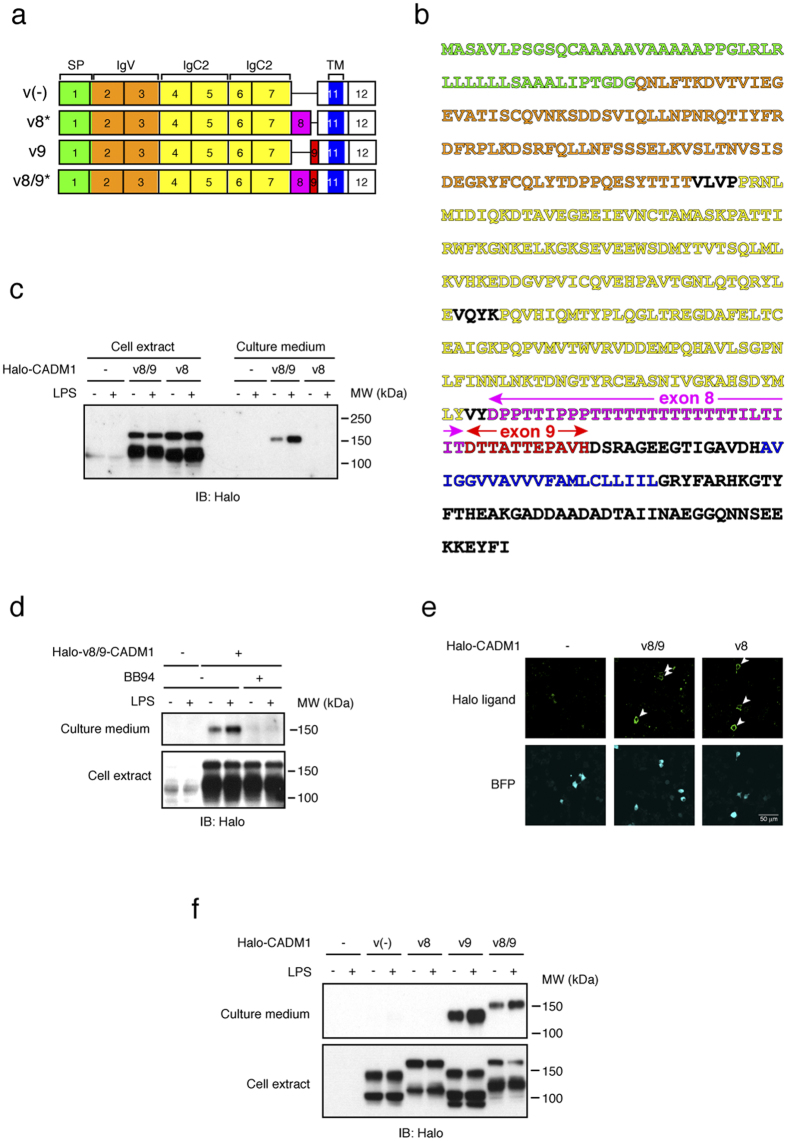
Alternative splicing of CADM1 gene gives rise to both shedding-susceptible and shedding-resistant CADM1 variant proteins. (**a**) Schematic diagrams of molecular structure of four splice variants of CADM1. Exons are indicated by black rectangles with numbers, and exon 8 and exon 9 are colored in magenta and red, respectively. Two splice variants expressing in Raw 264.7 cells, v8 and v8/9 CADM1, are indicated by asterisks. SP, signal peptide (green); IgV, immunoglobulin (Ig)-like variable-type domain (orange); IgC2, Ig-like constant 2-type domain (yellow); TM, transmembrane domain (blue). (**b**) Amino acid sequence of v8/9 CADM1. The characters are colored as in Fig. 1A. Amino acids encoded by exon 8 and exon 9 are indicated. (**c**) Raw 264.7 cells expressing N-terminally Halo-tagged v8 or v8/9 CADM1 were treated with (+) or without (−) 1 μg/ml LPS for 60 min. Both cell extracts (Cell extract) and culture supernatants (Culture medium) were subjected to Western blotting with an anti-Halo antibody. (**d**) Raw 264.7 cells expressing Halo-tagged v8/9 CADM1 were treated with LPS and/or 10 μM BB94 for 60 min as indicated, and cell extracts and culture supernatants were subjected to Western blotting. (**e**) Raw 264.7 cells expressing Halo-tagged v8 or v8/9 CADM1 were stained using cell-impermeable Alexa 488 HaloTag ligand (Halo ligand). Arrowheads indicate transfected BFP-positive cells (BFP). Bar, 50 μm. (**f**) Raw 264.7 cells expressing Halo-tagged CADM1 variant proteins were treated with LPS, and cell extracts and culture supernatants were subjected to Western blotting.

To confirm that the candidate shedding targets identified in this study are shed in LPS-stimulated Raw 264.7 cells, we focused upon CADM1 (also known as SynCAM1 (synaptic cell adhesion molecule 1) and TSLC1 (tumor suppressor in lung cancer 1)), a member of the immunoglobulin superfamily cell adhesion molecules^15^,^16^, since shedding of CADM1 has been reported previously^17^,^18^,^19^,^20^. CADM1 is encoded by 12 exons in the mouse, and 6 splice variants are generated through the combination of alternative exons 8/9/10^21^,^22^. As shown in Fig. 1a, the constitutive exons 6 and 7 encode the extracellular membrane-proximal Ig-like C2 domain, while the constitutive exon 11 encodes the transmembrane domain. Accordingly, alternative exons 8/9/10 encode the stalk region of CADM1, where shedding should occur. Thus, we amplified the coding sequence of CADM1 from a cDNA library of Raw 264.7 cells to evaluate the shedding of CADM1 variants expressed in the cells. We obtained two splice variants of CADM1, v8 and v8/9. V8 CADM1 contains exon 8 between the constitutive exons 7 and 11, while v8/9 CADM1 contains both exons 8 and 9 (Fig. 1a,b). We did not obtain any CADM1 variants containing exon 10, a minor exon. To examine the shedding of v8 and v8/9 CADM1 individually, Raw 264.7 cells expressing N-terminally Halo-tagged CADM1 variants were treated with or without LPS, and the cell extracts and culture media were subjected to Western blotting using an anti-Halo antibody. Unpredictably, Halo-tagged, soluble CADM1 with an apparent molecular weight corresponding to the entire extracellular domain was only detected in the culture medium of v8/9 CADM1 expressing cells, even though both CADM1 variant proteins were equally detected in the cell extracts (Fig. 1c). These observations indicate that v8/9 CADM1 is shedding-susceptible, while v8 CADM1 is shedding-resistant in Raw 264.7 cells. We found that v8/9 CADM1 is significantly more sensitive to shedding than v8 CADM1 in human small cell lung carcinoma SBC-5 cells and canine epithelial MDCK cells (Supplementary Fig. 2). The release of soluble v8/9 CADM1 was substantially increased by LPS and completely suppressed by BB94 (Fig. 1c,d), indicating that v8/9 CADM1 is shed by metalloprotease(s) in an LPS-activated manner.

In many cases, cell-surface localization of shedding targets is required for their shedding. We, therefore, evaluated the cell-surface localization of v8 and v8/9 CADM1 using a cell-impermeable HaloTag fluorescent ligand. We detected equal amounts of fluorescent signal on the surface of v8 and v8/9 CADM1 expressing cells (Fig. 1e), indicating that cell surface localization of CADM1 variants is not the determinant of their shedding susceptibility.

We next constructed two additional splice variants, v(-) and v9 CADM1, the former lacking all alternative exons and the latter containing only exon 9 (Fig. 1a), and evaluated their shedding susceptibility. There was no sign of shedding of v(-) CADM1 in the culture medium, whereas v9 CADM1 was shed as effectively as v8/9 CADM1 (Fig. 1f). Collectively, these observations demonstrate that the inclusion of only 11 amino acid residues encoded by exon 9 is necessary and sufficient for the shedding susceptibility of CADM1 variant proteins. It is noteworthy that the exon corresponding to mouse exon 9 is evolutionarily conserved from fish to human (Supplementary Fig. 3), emphasizing its functional significance.

### Alternative splicing can also give rise to both shedding-susceptible and shedding-resistant SIRPα variant proteins

**Figure 2:**
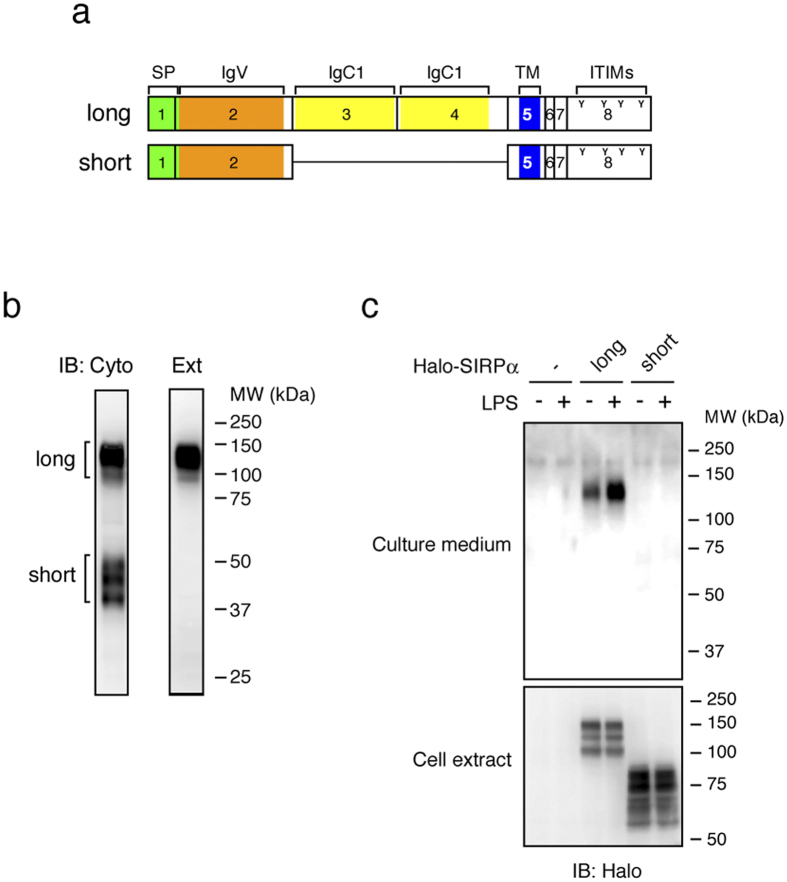
Alternative splicing of SIRPα gene also gives rise to both shedding-susceptible and shedding-resistant SIRPα variant proteins. (**a**) Schematic diagrams of molecular structure of two splice variants of SIRPα. Exons are indicated by black rectangles with numbers. IgC1, Ig-like constant 1-type domain (yellow); ITIMs, immunoreceptor tyrosine-based inhibitory motif. Y characters indicate tyrosine residues in ITIMs. (**b**) Raw 264.7 cell extracts were subjected to Western blotting using the antibody recognizing cytoplasmic domain of SIRPα (Cyto) or extracellular domain of SIRPα (Ext). (**c**) Raw 264.7 cells expressing Halo-tagged SIRPα variant proteins were treated with or without LPS for 60 min, and cell extracts and culture supernatants were subjected to Western blotting as in Fig. 1.

We searched for other shedding targets having multiple splice variants using Ensembl database (http://www.ensembl.org/) and found that SIRPα (signal-regulatory protein alpha), a member of the immunoglobulin superfamily inhibitory receptors^23^,^24^, has splice variants. We amplified the coding sequence of SIRPα from a cDNA library of Raw 264.7 cells and obtained two SIRPα splice variants (Fig. 2a). One encodes the well-studied 509-amino-acid SIRPα (hereafter called long SIRPα), and the other encodes the 291-amino-acid SIRPα (hereafter called short SIRPα). Long SIRPα is encoded by 8 exons, whereas short SIRPα lacks exons 3 and 4 which encode extracellular two Ig-like C1 domains (Fig. 2a). Western blotting of Raw 264.7 cell extract revealed that the antibody recognizing the cytoplasmic tail of SIRPα, which is common to both long and short SIRPα variants, detects ~130 kDa and ~40 kDa proteins, whereas the antibody recognizing the extracellular domain of SIRPα, which is largely absent in short SIRPα, only detects ~130 kDa protein (Fig. 2b). These observations indicate that the comparable amounts of both SIRPα variant proteins are expressed in Raw 264.7 cells.

We next evaluated the shedding susceptibility of long and short SIRPα using N-terminally Halo-tagged proteins. Whereas both SIRPα proteins were expressed in cell extracts to comparable extents, only soluble long SIRPα was released into the culture medium (Fig. 2c). These results support the notion that both shedding-susceptible and shedding-resistant variant proteins can be generated from a single gene via alternative splicing.

### ADAM17 is the responsible sheddase for both CADM1 and SIRPα

**Figure 3:**
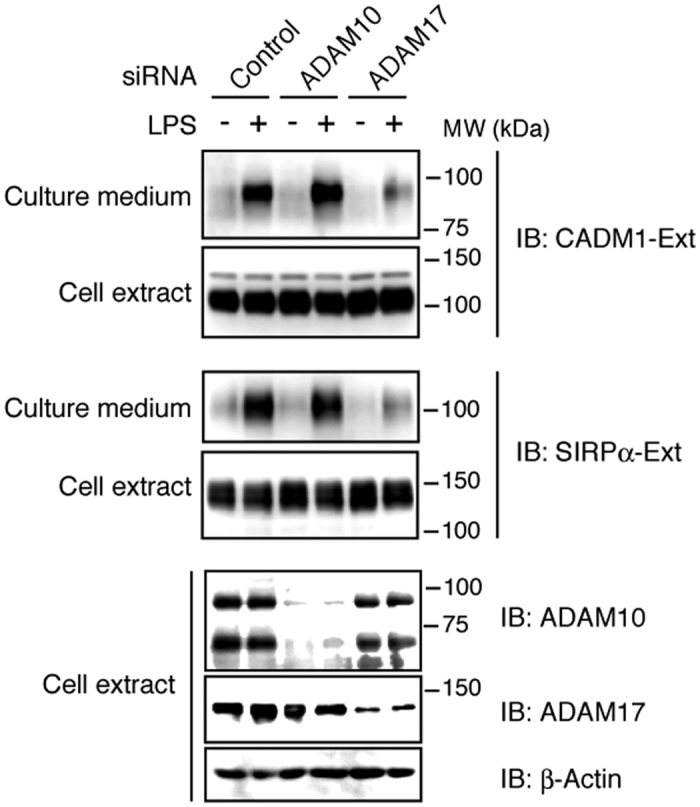
ADAM17 is the responsible sheddase for both CADM1 and SIRPα. Raw 264.7 cells transfected with siRNA against ADAM10 or ADAM17 as indicated were treated with or without LPS for 60 min, and cell extracts and culture supernatants were subjected to Western blotting using the indicated antibodies. Ext, extracellular domain.

Because two ADAM family metalloproteases, ADAM10 and ADAM17, execute the majority of reported shedding events^3^,^4^, we examined which ADAM is responsible for shedding of CADM1 and SIRPα. Western blotting using the antibody recognizing extracellular domain of CADM1 or SIRPα revealed that endogenous CADM1 and SIRPα were shed in LPS-stimulated Raw 264.7 cells (Fig. 3). The release of soluble CADM1 and SIRPα was significantly suppressed by knockdown of ADAM17 but not by that of ADAM10 (Fig. 3), indicating that ADAM17 is the responsible sheddase for CADM1 and SIRPα.

We found that the amount of v8/9 CADM1 mRNA is roughly 10-fold lower than that of v8 CADM1 mRNA in the resting state, and LPS treatment does not alter their relative abundance within the timeframe of shedding activation (within hours) (Supplementary Fig. 4). These observations indicate that CADM1 shedding is activated in an alternative splicing-independent manner in LPS-stimulated macrophages.

### Exon 9 does not encode the shedding cleavage site of CADM1

In contrast to SIRPα variant proteins, those of CADM1 have almost the same molecular structures, although the insertion of only 11 amino acids encoded by exon 9 completely converts their shedding susceptibility. We assumed that the 11 amino acids contain important factor(s) determining shedding susceptibility of membrane proteins, and thus analyzed in detail.

**Figure 4:**
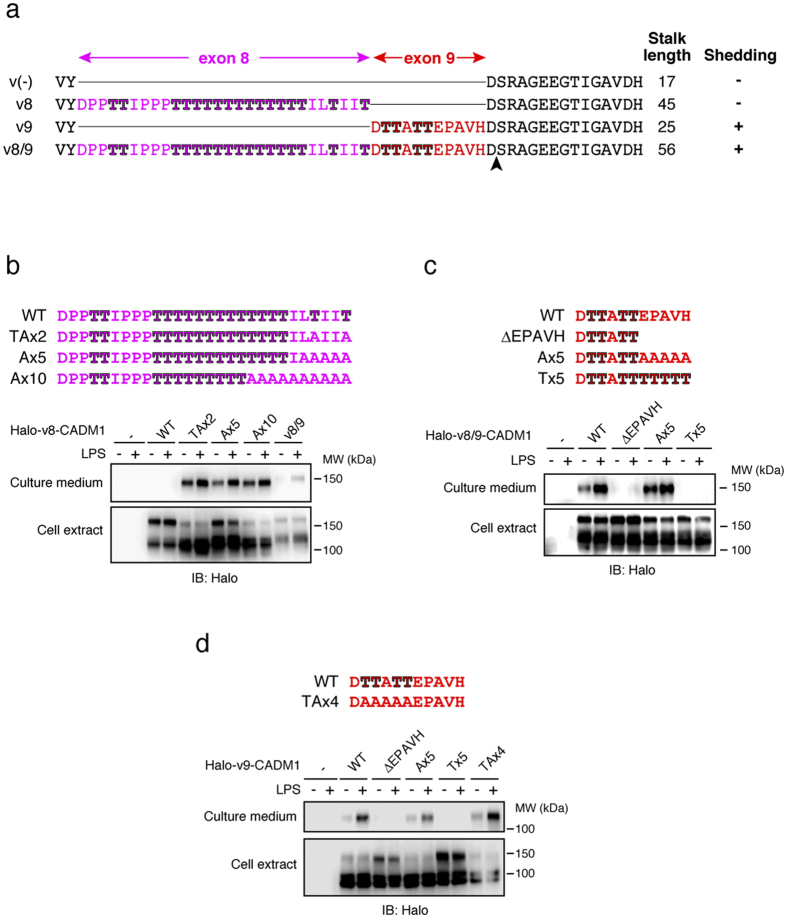
Exon 9 confers shedding susceptibility to CADM1 through five non-glycosylatable amino acids. (**a**) The amino acid sequences of stalk region (between membrane-proximal IgC2 domain and transmembrane domain) of CADM1 variant proteins. The amino acids encoded by exon 8 and exon 9 are colored in magenta and red, respectively. Threonine residues serving as potential *O*-glycosylation sites are highlighted. An arrowhead indicates the shedding cleavage site of v8/9 CADM1. (**b**–**d**) Raw 264.7 cells expressing Halo-tagged v8 CADM1 mutants (**b**), v8/9 CADM1 mutants (**c**), or v9 CADM1 mutants (**d**) were treated with LPS for 60 min, and cell extracts and culture supernatants were subjected to Western blotting. The amino acid sequences of substitution and deletion mutants are indicated above.

Since both scissile bond sequence and sufficient stalk length are considered to be required for shedding susceptibility of membrane proteins, we first examined whether exon 9 encodes the shedding cleavage site of CADM1. After confirming that v8/9 CADM1 is significantly more sensitive to shedding than v8 CADM1 in HEK 293 cells, the cytoplasmic PA-tagged v8/9 CADM1 was highly expressed in HEK 293 cells, and the ~15 kDa membrane-remaining shedding product was affinity purified (Supplementary Fig. 5a,b). The N-terminal sequencing revealed that it starts with Ser-Xaa-Ala-Xaa-Glu-Glu (Supplementary Fig. 5c), indicating that v8/9 CADM1 is cleaved between aspartic acid 374 and serine 375, which reside immediately downstream of the amino acid sequence encoded by exon 9 (Fig. 4a, arrowhead). It was previously reported that ADAM17 characteristically prefers proline residue at P5 position (5 amino acids upstream of the cleavage site)^6^, which is consistent with the identified cleavage site, suggesting the reliability of our analysis. These observations indicate that not exon 9 but exon 11 encodes the shedding cleavage site of CADM1.

### Exon 8 encodes many *O*-glycosylatable threonine residues interfering with CADM1 shedding

Sufficient stalk length is also required for shedding susceptibility of membrane proteins, and the amino acid sequence encoded by exon 8 and 9 both reside in the stalk region of CADM1 (Fig. 4a). However, although exon 8 is longer than exon 9, the inclusion of exon 8 did not confer shedding susceptibility to CADM1 (Figs 1f and 4a), suggesting the existence of structural barrier(s) within the amino acid sequence encoded by exon 8. Exon 8 encodes many threonine residues (60.7%, 17/28), most of which are predicted to be *O*-glycosylated by NetOGlyc 4.0 prediction server (http://www.cbs.dtu.dk/services/NetOGlyc/)^25^. Even though it is impossible to accurately predict all *O*-glycosylated residues using an algorithm, the analysis results suggest that a large percentage of threonine residues encoded by exon 8 are *O*-glycosylated. Thus, we examined whether *O*-glycans attached to the threonine residues encoded by exon 8 interfere with CADM1 shedding. Alanine substitutions of two threonine residues which reside immediately upstream of the shedding cleavage site conferred shedding susceptibility to v8 CADM1 (Fig. 4b, TAx2). Single alanine substitution of the most cleavage-site-proximal threonine residue moderately conferred shedding susceptibility to v8 CADM1 (Supplementary Fig. 6). Additional alanine substitutions did not potentiate further shedding (Fig. 4b, Ax10). These observations suggest that *O*-glycans attached to the threonine residues adjacent to the shedding cleavage site interfere with shedding of v8 CADM1, and the insertion of six non-glycosylatable amino acids between the shedding cleavage site and interfering *O*-glycans is sufficient to confer shedding susceptibility to CADM1.

### Exon 9 confers shedding susceptibility to CADM1 through the insertion of five non-glycosylatable amino acids

Exon 9 encodes four threonine residues in its N-terminal half and five non-glycosylatable amino acids in its C-terminal half (Fig. 4a). Since the five non-glycosylatable amino acids reside immediately upstream of the shedding cleavage site (Fig. 4a), we speculated that exon 9 confers shedding susceptibility to CADM1 through the insertion of the five amino acids, and thus examined this possibility. Deletion of the five amino acids completely abolished shedding of v8/9 CADM1 (Fig. 4c, ΔEPAVH), while insertion of five alanine residues restored the shedding of the deletion mutant (Fig. 4c, Ax5), however, the insertion of five threonine residues failed to restore (Fig. 4c, Tx5). We obtained the equivalent results using v9 CADM1 (Fig. 4d). Meanwhile, alanine substitutions of four N-terminal threonine residues had no effect on the shedding of v9 CADM1 (Fig. 4d, TAx4). Collectively, these observations indicate that exon 9 confers shedding susceptibility to CADM1 through the insertion of five non-glycosylatable amino acids independently with their sequences.

### Threonine residues encoded by exons 8 and 9 are *O*-glycosylated

**Figure 5:**
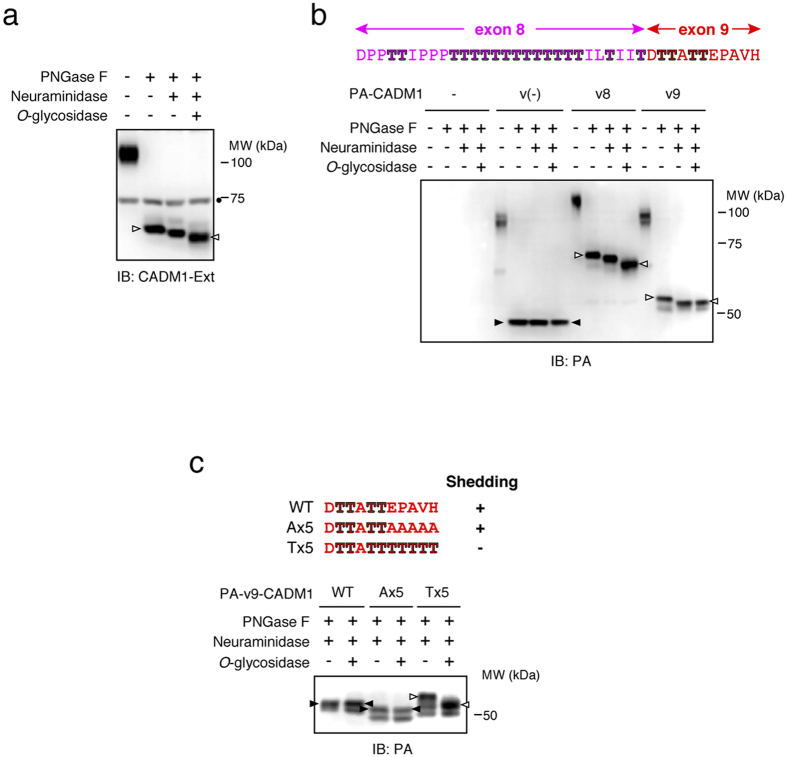
Threonine residues encoded by both exon 8 and exon 9 are certainly *O*-glycosylated. (**a**) The extract of unstimulated Raw 264.7 cell was treated with indicated glycosydases and subjected to Western blotting with an anti-CADM1 antibody. Closed circle indicates a non-specific band. (**b**,**c**) PA-tagged v(-), v8 and v9 CADM1 (**b**) or substitution mutants of v9 CADM1 (**c**) were expressed in Raw 264.7 cells, and the extracts of expressing cells were treated with indicated glycosydases and subjected to Western blotting with an anti-PA antibody. Black triangles indicate the CADM1 proteins whose molecular weights were not reduced by neuraminidase or *O*-glycosidase, whereas white triangles indicate the CADM1 proteins whose molecular weights were reduced. The amino acid sequences of exon 8 and exon 9 of wild-type and substitution mutants are indicated above.

We examined whether endogenous CADM1 is *O*-glycosylated. Removal of *N*-glycans by PNGase F (peptide *N*-glycosidase F) treatment significantly decreased the molecular weight of endogenous CADM1, indicating that endogenous CADM1 is highly *N*-glycosylated as reported previously^22^ (Fig. 5a, left triangle). After removal of *N*-glycans, both neuraminidase and *O*-glycosidase treatment further decreased the molecular weight of endogenous CADM1 (Fig. 5a, right triangle), indicating that endogenous CADM1 is *O*-glycosylated.

We next examined whether threonine residues encoded by exons 8 and 9 are *O*-glycosylated using three CADM1 splice variants, v(-), v8, and v9. After removal of *N*-glycans, neither neuraminidase nor *O*-glycosidase treatment decreased the molecular weight of v(-) CADM1 (Fig. 5b, black triangles). On the other hand, the molecular weight of both v8 and v9 CADM1 was decreased by neuraminidase treatment, and the molecular weight of v8 CADM1 was further decreased by *O*-glycosidase treatment (Fig. 5b, white triangles). These observations indicate that threonine residues encoded by exons 8 and 9 are *O*-glycosylated, and shedding-resistant v8 CADM1 is highly *O*-glycosylated relative to shedding-susceptible v9 CADM1.

We found that the *O*-glycosylation level of endogenous CADM1 remains largely unchanged in Raw 264.7 cells treated with LPS for an hour (data not shown), suggesting that the change of *O*-glycosylation does not participate in the activation of CADM1 shedding.

### The substituted threonine residues that interfere with shedding of v9 CADM1 are highly *O*-glycosylated

We further compared *O*-glycosylation level of v9 CADM1 mutants described in Fig. 4d to examine the relationship between *O*-glycosylation and shedding susceptibility. After treatment with both PNGase F and neuraminidase, *O*-glycosidase treatment did not decrease the molecular weight of shedding-susceptible Ax5 mutant of v9 CADM1, similarly to that of wild-type v9 CADM1 (Fig. 5c, black triangles). In contrast, *O*-glycosidase treatment substantially decreased the molecular weight of the shedding-resistant Tx5 mutant of v9 CADM1 (Fig. 5c, white triangles), indicating that the substituted threonine residues that interfere with shedding of v9 CADM1 are highly *O*-glycosylated.

## Discussion

In this study, we provide evidence that the shedding susceptibility of an adhesion molecule, CADM1, is strictly regulated by two different and independent modification mechanisms, alternative splicing and *O*-glycosylation. These observations substantially expand our knowledge about molecular mechanisms ensuring high selectivity of naturally occurring shedding.

First, we show that *O*-glycosylation of CADM1 interferes with its ADAM17-mediated shedding. *O*-glycosylation is found on the majority of secreted and membrane proteins and often in their linker and stalk regions^25^,^26^. It is widely accepted that *O*-glycosylation of secreted proteins prevents processing by furin family proprotein convertases^26^,^27^, however, whether *O*-glycosylation of membrane proteins generally prevents ADAM-mediated shedding is somewhat controversial. It has been reported that some physiologically “shedding-resistant” membrane proteins, including LDL (low density lipoprotein) receptor family members^28^,^29^,^30^ and IL-2 receptor alpha chain^31^, are released into culture medium when their *O*-glycosylation is artificially reduced, suggesting that *O*-glycosylation prevents these proteins from aberrant shedding under physiological conditions, even though it is not clear which proteases are responsible for the shedding. In addition, it has been reported that the shedding of TNFα is potentiated by the deletion of GalNAc-T2 *O*-glycosylation enzyme both in cell line and *in vivo*^32^. Even though these cell-based and *in vivo* studies indicate that *O*-glycosylation generally prevents shedding, the results obtained from *in vitro* cleavage assays are somewhat confusing. *O*-glycosylation of synthetic peptides reduces cleavage by recombinant soluble ADAM in many cases, but potentiates it in a few cases^32^,^33^,^34^. In this study, we show that *O*-glycans attached to the threonine residues adjacent to the shedding cleavage site of CADM1 significantly prevent its ADAM17-mediated shedding, and provide clear evidence that *O*-glycosylation of physiological ADAM17 substrate prevents its shedding. We speculate that *O*-glycosylation of membrane proteins generally prevents their ADAM-mediated shedding on the cell surface, and that *in vitro* cleavage assay is not suitable to evaluate ADAM-mediated shedding in which both enzyme and substrate are tethered to the same membrane and thus conformationally restrained.

**Figure 6:**
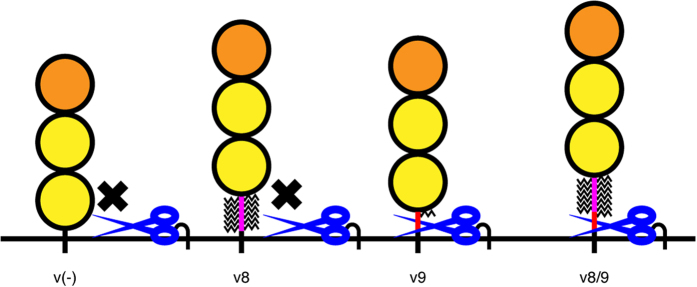
Shedding susceptibility of CADM1 is determined by both alternative splicing and *O*-glycosylation. Schematic diagram of the structure of stalk region of CADM1 variant proteins and their shedding susceptibility. Orange, IgV domain; yellow, IgC2 domain; magenta, amino acids encoded by exon 8; red, amino acids encoded by exon 9; broken lines, *O*-glycans; blue scissors, ADAM17.

We further elucidate how the small alternative exon, exon 9, confers shedding susceptibility to CADM1 in the presence of interfering *O*-glycans. Among 11 amino acids encoded by exon 9, five C-terminal non-glycosylatable amino acids which reside immediately upstream of the shedding cleavage site are indispensable for conferring shedding susceptibility, and these amino acids can be substituted to alanine residues without affecting the shedding susceptibility of exon 9-containing CADM1 variants. These results indicate that “a stretch of five non-glycosylated amino acids” is sufficient to confer shedding susceptibility, that is, exon 9 confers shedding susceptibility to CADM1 by increasing the distance between interfering *O*-glycans and the shedding cleavage site (Fig. 6). Therefore, we would like to propose here that the term “stalk length”, a determinant of shedding susceptibility, should be redefined as the length between the cell surface and membrane-proximal extracellular domain “or membrane-proximal *O*-glycosylated amino acid”. Applying this new concept of stalk length, our results further indicate that as few as 5-amino-acid extension of stalk length can switch the shedding susceptibility of CADM1. These observations suggest that membrane proteins have their own “threshold stalk length”, and only membrane proteins having stalk region longer than that are susceptible to shedding. In other words, this study emphasizes the role of stalk length in determining shedding susceptibility.

It was reported that ErbB4 receptor tyrosine kinase has both shedding-susceptible (so-called “JM-a”) and shedding-resistant (“JM-b”) splice variant proteins generated by the switching of two mutually exclusive exons (75 bp and 45 bp), both of which encode its stalk region^35^,^36^. Since JM-a variant protein is cleaved within the amino acid sequence encoded by the alternative exon^37^, it is supposed that the exon confers shedding susceptibility to ErbB4 by the insertion of scissile bond sequence. However, our present results support the possibility that the difference in stalk length determines shedding susceptibility of ErbB4 variant proteins, because the stalk region of JM-a variant is 10 amino acids longer than that of JM-b variant. We found that another shedding target identified in this study, CD166, has splice variants generated by the inclusion/skipping of a 39-bp alternative exon encoding its stalk region, demonstrating that multiple shedding target-coding genes contain small alternative exons encoding their stalk regions. These observations raise the intriguing possibility that shedding susceptibility of a certain number of membrane proteins is regulated by splicing of alternative exons encoding their stalk regions, which only changes their stalk length. Shedding susceptibility of deletion/substitution mutants of JM-a variant and CD166 splice variants should be evaluated to validate this possibility.

In this study, we also show that alternative splicing of SIRPα gene generates both shedding-susceptible (long) and shedding-resistant (short) variant proteins. Consistent with the notion that the stalk length is a determinant of shedding susceptibility, long SIRPα has a longer stalk region than short SIRPα, however, the long SIRPα mutant containing the stalk region of short SIRPα was still susceptible to shedding (data not shown). Unlike CADM1, SIRPα variant proteins have completely different extracellular domains. We, therefore, speculate that the extracellular domain of membrane proteins is a determinant of their threshold stalk length. SIRPα variant proteins have no serine/threonine residues predicted to be *O*-glycosylated in their stalk regions, ruling out the involvement of *O*-glycosylation in the determination of their shedding susceptibility.

Since v8/9 CADM1 is significantly more sensitive to shedding than v8 CADM1 even in SBC-5, MDCK, and HEK 293 cells, we consider that the shedding susceptibility of CADM1 is determined by exon 9 in many cell types and species. We have previously reported that the relative abundance of exon 9-containing CADM1 variant mRNAs is strictly regulated in a tissue- and disease-specific manner^22^. Similar to Raw 264.7 cells, a large amount of v8 CADM1 mRNA and only a small but detectable amount of v8/9 CADM1 mRNA are expressed in nearly all mouse tissues. In contrast, considerable amounts of v9 and v8/9 CADM1 mRNAs, both encoding shedding-susceptible variant proteins, are dominantly expressed in the mouse testis. Since the most prominent phenotype of CADM1-deficient mice is male infertility with the arrest of spermatogenesis in the testis^38^,^39^, CADM1 might play important roles in spermatogenesis in its shedding-dependent manner. On the other hand, CADM1 is a tumor suppressor gene whose expression is frequently lost in various human carcinomas^15^,^40^, but substantial and nearly equal amounts of v8 and v8/9 CADM1 mRNAs are expressed in a neuroendocrine subtype of lung cancer^22^. Shedding can not only decrease the amount of full-length proteins but also generate the cleaved intracellular domains that translocate to the nucleus and regulate gene expression. It is, therefore, interesting to speculate that shedding-susceptible CADM1 variant proteins can function as signal-transducing adhesion molecules. Future studies are needed to elucidate how a high proportion of shedding-susceptible CADM1 variant proteins contributes to proper spermatogenesis in testis and malignant transformation in specific types of lung cancer.

Taken together, this study’s results clearly show that shedding susceptibility of cell surface protein can be regulated at two different levels of its biosynthesis pathway, alternative splicing occurring in the nucleus and *O*-glycosylation occurring in the Golgi apparatus. These two modification mechanisms enable the simultaneous expression of both shedding-susceptible and shedding-resistant variant proteins with different ratios in a cell-type-specific manner, thereby contributing to high selectivity of naturally occurring shedding. We believe that the very existence of these elaborate mechanisms strongly indicate the physiological importance of ectodomain shedding.

## Methods

### Cell line, transfection, and sample preparation for Western blotting

A murine macrophage cell line, Raw 264.7, was cultured in high glucose DMEM supplemented with 10% fetal bovine serum, 50 μM 2-mercaptoethanol, and antibiotics. Transfections were performed with FuGENE HD (Promega, Madison, WI) in general and with a NEPA21 electroporator (NEPA GENE, Chiba, Japan) in the siRNA knockdown and glycosidase treatment experiments. For Western blotting, cell extracts and proteins of conditioned culture media were prepared as described previously^13^.

### Antibodies, expression plasmids, siRNAs, chemicals, and glycosidases

Antibodies were purchased from Promega (anti-HaloTag, G9211), MBL (Nagoya, Japan, anti-CADM1, 3E1), BioLegend (San Diego, CA, anti-SIRPα-Ext, P84), ProSci (Poway, CA, anti-SIRPα-C, 1125), Abcam (Cambridge, UK, anti-ADAM10, ab39177 and anti-ADAM17, ab39162), Santa Cruz (Dallas, TX, anti-β-actin, sc-81178), and WAKO (Osaka, Japan, anti-PA tag, NZ-1). The N-terminal-tagged expression vectors were constructed as described previously^13^. The coding sequences of CADM1 and SIRPα variant proteins were obtained by PCR from cDNA library constructed from untreated Raw 264.7 cells. V(-), v9, and mutants of CADM1 were constructed using a PCR-based method. The coding sequences of CADM1 and SIRPα variant proteins were subcloned into N-terminal-tagged expression vectors after removal of their own signal sequences (Met1-Gly47 and Met1-Gly31, respectively). PA tag (WAKO) was inserted between Gly-443 and Gly-444 of v8/9-CADM1 using a PCR-based method to construct cytoplasmic PA-tagged CADM1. All siRNAs were purchased from Thermo Fisher Scientific (Waltham, MA). LPS was purchased from Sigma-Aldrich (Saint-Louis, MO). BB94 was provided by Vernalis (Berkshire, UK). PNGase F, neuraminidase, and *O*-glycosidase were purchased from New England Biolabs (Ipswich, MA).

### Purification of *N*-glycosylated peptides from conditioned medium of SILAC cultured cells

DMEM medium for SILAC (Thermo Fisher Scientific) was supplemented with 10% dialyzed fetal bovine serum (Thermo Fisher Scientific), 50 μM 2-mercaptoethanol, and 100 mg/l L-Lysine-^12^C_6_ and L-Arginine-^12^C_6_,^14^N_4_ (Sigma-Aldrich) in the light medium, or L-Lysine-^13^C_6_ and L-Arginine-^13^C_6_,^15^N_4_ (Silantes, Munchen, Germany) in the heavy medium, respectively. Raw 264.7 cells were grown in light or heavy medium for 1 week to metabolically label all expressing proteins. Twenty million cells were washed twice with serum-free medium and seeded into a single 10-cm culture dish filled with serum-free light or heavy medium in the day before sampling. Cells were cultured in fresh serum-free light or heavy medium containing 1 μg/ml LPS with or without 20 μM BB94 for 60 min, and conditioned media were collected, centrifuged, and mixed. To enrich peptides derived from membrane proteins, *N*-glycosylated peptides were concentrated from the media according to the method described previously^41^,^42^ with slight modifications.

The media were acidified by 50 mM acetic acid buffer (pH 5) and oxidized by 10 mM NaIO_4_ at room temperature for 30 min. Proteins were precipitated with methanol/chloroform solution and suspended in a buffer containing 8 M urea and 400 mM NH_4_HCO_3_. Approximately 15 μg of protein was obtained from 2 × 10^7^ cells, and 100 μg was reduced with 10 mM DTT at room temperature for 30 min, alkylated with 20 mM iodoacetamide for 30 min in the dark, diluted 4-fold with distilled water, and digested by trypsin (Promega) at an enzyme:substrate ratio of 1:50 (w/w) at 37 °C overnight. The digested peptides were incubated with Affi-Gel Hz hydrazide gel in Affi-Gel Hz coupling buffer (BioRad, Hercules, CA) at room temperature overnight. After sequential washing with 1.5 M NaCl, 80% acetonitrile, CH_3_OH, distilled water, and 50 mM NH_4_HCO_3_, the resin was incubated with 1000 U PNGase F in 50 mM NH_4_HCO_3_ at 37 °C overnight. The beads were centrifuged and the supernatant was divided into three parts and subjected to liquid chromatography tandem mass spectrometry (LC-MS/MS) in triplicate.

### Identification and quantification of *N*-glycosylated peptides

Peptides were analyzed by nano-LC-ESI-MS/MS using an UltiMate 3000 nano-LC system (Thermo Fisher Scientific) linked to a QSTAR Elite hybrid LC-MS/MS system (AB SCIEX, Framingham, MA). Samples were loaded onto an L-column 2 ODS 0.075 mm × 100 mm, 3 μm (CERI, Tokyo, Japan) and separated at 300 nl/min with 33 min linear gradients from 6 to 34% acetonitrile in 0.1% formic acid. Identification and quantification of peptides were performed using ProteinPilot software version 4.0.8085 (AB SCIEX). The following search parameters were selected: sample type (SILAC (Lys + 8, Arg + 10)), cys alkylation (iodoacetamide), digestion (trypsin), instrument (QSTAR Elite ESI), special factors (default), species (mouse), ID focus (biological modifications), databases (UniProt_Sprot_contaminants + iso_08092010 database), and search effort (thorough). The accuracy tolerance for both peptides and peptide fragments was set to 0.10 Da with a 95% confidence threshold. The false discovery rate (FDR) analysis was done using the integrated tools in ProteinPilot. Peptides containing deamidation(s) of asparagine residue(s) indicating former *N*-glycosylation and having H/L ratio ranging from 0.1 to 10 were selected, and the geometrical mean of H/L ratio was calculated for each protein.

### Cell surface staining of Halo-tagged proteins

Raw 264.7 cells expressing both N-terminally Halo-tagged CADM1 variant proteins and BFP (Evrogen, Moscow, Russia) were stained with cell-impermeable HaloTag Alexa Fluor 488 Ligand (Promega) according to the manufacturers’ instructions. Fluorescence images were taken under a FLUOVIEW FV10i-DOC confocal fluorescence microscope (Olympus, Tokyo, Japan).

### Glycosidase treatment

Cell extracts were digested by PNGase F, neuraminidase, and/or *O*-glycosidase according to the manufacturers’ instructions. To detect subtle decrease of molecular weights, 12-amino-acid PA tag was used instead of ~33 kDa Halo-tag in the experiments of glycosidase treatment.

## Additional Information

**How to cite this article:** Shirakabe, K. *et al*. Mechanistic insights into ectodomain shedding: susceptibility of CADM1 adhesion molecule is determined by alternative splicing and *O*-glycosylation. *Sci. Rep.* **7**, 46174; doi: 10.1038/srep46174 (2017).

**Publisher's note:** Springer Nature remains neutral with regard to jurisdictional claims in published maps and institutional affiliations.

## Supplementary Material

Supplementary Information

## Figures and Tables

**Table 1:** Candidate shedding targets of LPS-stimulated Raw 264.7 macrophages identified by proteomic screening using SILAC.

	Protein name	Gene name	Accession number	Mean of H/L ratio	No. of quantified peptides	Coverage	Function	TM domain	Ig domain
Previously identified	M-CSF receptor	CSF1R	P09581	1.68	43	11.05	Tyrosine kinase receptor	1	C2 × 5
	MHC class I α chain D	HA12	P01900	3.10	7	9.86	Antigen presentation	1	C1 × 1
	Semaphorin 4D	SEM4D	O09126	5.29	3	3.14	Chemorepellent receptor	1	C2 × 1
	MHC class I α chain L	HA1L	P01897	5.45	2	11.05	Antigen presentation	1	C1 × 1
Newly identified	CADM1	CADM1	Q8R5M8	3.44	15	17.54	Cell adhesion molecule	1	V × 1, C2 × 2
	SIRPα	SHPS1	P97797	2.37	14	20.86	Inhibitory CD47 receptor	1	V × 1, C1 × 2
	RANK	TNR11	O35305	2.79	7	2.24	TNF receptor superfamily	1	—
	MHC class I α chain K	HA1D	P01902	2.42	7	3.26	Antigen presentation	1	C1 × 1
	Nectin-1	PVRL1	Q9JKF6	6.60	5	13.79	Cell adhesion molecule	1	V × 1, C2 × 2
	CD166	CD166	Q61490	2.98	5	6.17	Cell adhesion molecule	1	V × 2, C2 × 3
	Basigin	BASI	P18572	1.87	3	4.63	Mannose binding	1	C2 × 1, V × 1
	CD47	CD47	Q61735	2.90	2	12.87	SIRPα ligand	5	V × 1
	CD200 receptor 1	MO2R1	Q9ES57	2.28	2	15.95	Inhibitory CD200 receptor	1	V × 1, C2 × 1
	Endothelial protein C receptor	EPCR	Q64695	5.85	1	11.98	Protein C receptor	1	—
	TNF receptor 1	TNR1A	P25118	4.16	1	5.51	TNF receptor	1	—
	TIMD3	HAVR2	Q8VIM0	2.79	1	9.25	Macrophage activation	1	V × 1
	ZIP6	S39A6	Q8C145	2.39	1	1.67	Zinc ion transporter	8	—
	Semaphorin 4 C	SEM4C	Q64151	2.11	1	1.44	Chemorepellent receptor	1	C2 × 1

## References

[b1] Seals D. F. & Courtneidge S. A. The ADAMs family of metalloproteases: multidomain proteins with multiple functions. Genes & development 17, 7–30 (2003). 12514095 10.1101/gad.1039703

[b2] Blobel C. P. ADAMs: key components in EGFR signalling and development. Nature reviews Molecular cell biology 6, 32–43 (2005). 15688065 10.1038/nrm1548

[b3] Reiss K. & Saftig P. The “a disintegrin and metalloprotease” (ADAM) family of sheddases: physiological and cellular functions. Seminars in cell & developmental biology 20, 126–137 (2009). 19049889 10.1016/j.semcdb.2008.11.002

[b4] Huovila A. P., Turner A. J., Pelto-Huikko M., Karkkainen I. & Ortiz R. M. Shedding light on ADAM metalloproteinases. Trends in biochemical sciences 30, 413–422 (2005). 15949939 10.1016/j.tibs.2005.05.006

[b5] Brown M. S., Ye J., Rawson R. B. & Goldstein J. L. Regulated intramembrane proteolysis: a control mechanism conserved from bacteria to humans. Cell 100, 391–398 (2000). 10693756 10.1016/s0092-8674(00)80675-3

[b6] Caescu C. I., Jeschke G. R. & Turk B. E. Active-site determinants of substrate recognition by the metalloproteinases TACE and ADAM10. The Biochemical journal 424, 79–88 (2009). 19715556 10.1042/BJ20090549 PMC2774824

[b7] Yamazaki S. . Mice with defects in HB-EGF ectodomain shedding show severe developmental abnormalities. The Journal of cell biology 163, 469–475 (2003). 14597776 10.1083/jcb.200307035 PMC2173637

[b8] Hinkle C. L. . Selective roles for tumor necrosis factor alpha-converting enzyme/ADAM17 in the shedding of the epidermal growth factor receptor ligand family: the juxtamembrane stalk determines cleavage efficiency. The Journal of biological chemistry 279, 24179–24188 (2004). 15066986 10.1074/jbc.M312141200

[b9] Hattori M., Osterfield M. & Flanagan J. G. Regulated cleavage of a contact-mediated axon repellent. Science 289, 1360–1365 (2000). 10958785 10.1126/science.289.5483.1360

[b10] Vecchi M., Baulida J. & Carpenter G. Selective cleavage of the heregulin receptor ErbB-4 by protein kinase C activation. The Journal of biological chemistry 271, 18989–18995 (1996). 8702564 10.1074/jbc.271.31.18989

[b11] Migaki G. I., Kahn J. & Kishimoto T. K. Mutational analysis of the membrane-proximal cleavage site of L-selectin: relaxed sequence specificity surrounding the cleavage site. The Journal of experimental medicine 182, 549–557 (1995). 7543142 10.1084/jem.182.2.549 PMC2192138

[b12] Shirakabe K., Hattori S., Seiki M., Koyasu S. & Okada Y. VIP36 protein is a target of ectodomain shedding and regulates phagocytosis in macrophage Raw 264.7 cells. The Journal of biological chemistry 286, 43154–43163 (2011). 22016386 10.1074/jbc.M111.275586 PMC3234858

[b13] Shirakabe K., Shibagaki Y., Yoshimura A., Koyasu S. & Hattori S. A proteomic approach for the elucidation of the specificity of ectodomain shedding. Journal of proteomics 98, 233–243 (2014). 24456812 10.1016/j.jprot.2014.01.012

[b14] Ong S. E. . Stable isotope labeling by amino acids in cell culture, SILAC, as a simple and accurate approach to expression proteomics. Molecular & cellular proteomics: MCP 1, 376–386 (2002). 12118079 10.1074/mcp.m200025-mcp200

[b15] Murakami Y. Involvement of a cell adhesion molecule, TSLC1/IGSF4, in human oncogenesis. Cancer science 96, 543–552 (2005). 16128739 10.1111/j.1349-7006.2005.00089.x PMC11158471

[b16] Takai Y., Miyoshi J., Ikeda W. & Ogita H. Nectins and nectin-like molecules: roles in contact inhibition of cell movement and proliferation. Nature reviews Molecular cell biology 9, 603–615 (2008). 18648374 10.1038/nrm2457

[b17] Mimae T. . Increased ectodomain shedding of lung epithelial cell adhesion molecule 1 as a cause of increased alveolar cell apoptosis in emphysema. Thorax 69, 223–231 (2014). 24092566 10.1136/thoraxjnl-2013-203867 PMC3933066

[b18] Moiseeva E. P., Leyland M. L. & Bradding P. CADM1 is expressed as multiple alternatively spliced functional and dysfunctional isoforms in human mast cells. Molecular immunology 53, 345–354 (2013). 23063768 10.1016/j.molimm.2012.08.024 PMC3550521

[b19] Nagara Y. . Tumor suppressor cell adhesion molecule 1 (CADM1) is cleaved by a disintegrin and metalloprotease 10 (ADAM10) and subsequently cleaved by gamma-secretase complex. Biochemical and biophysical research communications 417, 462–467 (2012). 22172944 10.1016/j.bbrc.2011.11.140

[b20] Tanabe Y., Kasahara T., Momoi T. & Fujita E. Neuronal RA175/SynCAM1 isoforms are processed by tumor necrosis factor-alpha-converting enzyme (TACE)/ADAM17-like proteases. Neuroscience letters 444, 16–21 (2008). 18718504 10.1016/j.neulet.2008.08.023

[b21] Biederer T. Bioinformatic characterization of the SynCAM family of immunoglobulin-like domain-containing adhesion molecules. Genomics 87, 139–150 (2006). 16311015 10.1016/j.ygeno.2005.08.017

[b22] Kikuchi S. . Expression of a splicing variant of the CADM1 specific to small cell lung cancer. Cancer science 103, 1051–1057 (2012). 22429880 10.1111/j.1349-7006.2012.02277.x PMC7685078

[b23] Barclay A. N. & Brown M. H. The SIRP family of receptors and immune regulation. Nature reviews Immunology 6, 457–464 (2006). 10.1038/nri1859 16691243

[b24] Matozaki T., Murata Y., Okazawa H. & Ohnishi H. Functions and molecular mechanisms of the CD47-SIRPalpha signalling pathway. Trends in cell biology 19, 72–80 (2009). 19144521 10.1016/j.tcb.2008.12.001

[b25] Steentoft C. . Precision mapping of the human O-GalNAc glycoproteome through SimpleCell technology. The EMBO journal 32, 1478–1488 (2013). 23584533 10.1038/emboj.2013.79 PMC3655468

[b26] Schjoldager K. T. & Clausen H. Site-specific protein *O*-glycosylation modulates proprotein processing - deciphering specific functions of the large polypeptide GalNAc-transferase gene family. Biochimica et biophysica acta 1820, 2079–2094 (2012). 23022508 10.1016/j.bbagen.2012.09.014

[b27] Schjoldager K. T. . A systematic study of site-specific GalNAc-type *O*-glycosylation modulating proprotein convertase processing. The Journal of biological chemistry 286, 40122–40132 (2011). 21937429 10.1074/jbc.M111.287912 PMC3220544

[b28] Kingsley D. M., Kozarsky K. F., Segal M. & Krieger M. Three types of low density lipoprotein receptor-deficient mutant have pleiotropic defects in the synthesis of N-linked, O-linked, and lipid-linked carbohydrate chains. The Journal of cell biology 102, 1576–1585 (1986). 3700466 10.1083/jcb.102.5.1576 PMC2114220

[b29] Kozarsky K., Kingsley D. & Krieger M. Use of a mutant cell line to study the kinetics and function of O-linked glycosylation of low density lipoprotein receptors. Proceedings of the National Academy of Sciences of the United States of America 85, 4335–4339 (1988). 3380796 10.1073/pnas.85.12.4335 PMC280423

[b30] May P., Bock H. H., Nimpf J. & Herz J. Differential glycosylation regulates processing of lipoprotein receptors by gamma-secretase. The Journal of biological chemistry 278, 37386–37392 (2003). 12871934 10.1074/jbc.M305858200

[b31] Karabasheva D., Cole N. B. & Donaldson J. G. Roles for trafficking and *O*-linked glycosylation in the turnover of model cell surface proteins. The Journal of biological chemistry 289, 19477–19490 (2014). 24891503 10.1074/jbc.M114.564666 PMC4094058

[b32] Goth C. K., Halim A., Khetarpal S. A., Rader D. J., Clausen H. & Schjoldager K. T. A systematic study of modulation of ADAM-mediated ectodomain shedding by site-specific O-glycosylation. Proceedings of the National Academy of Sciences of the United States of America 112, 14623–14628 (2015). 26554003 10.1073/pnas.1511175112 PMC4664366

[b33] Minond D. . Discovery of novel inhibitors of a disintegrin and metalloprotease 17 (ADAM17) using glycosylated and non-glycosylated substrates. The Journal of biological chemistry 287, 36473–36487 (2012). 22927435 10.1074/jbc.M112.389114 PMC3476313

[b34] Boskovski M. T. . The heterotaxy gene GALNT11 glycosylates Notch to orchestrate cilia type and laterality. Nature 504, 456–459 (2013). 24226769 10.1038/nature12723 PMC3869867

[b35] Elenius K. . A novel juxtamembrane domain isoform of HER4/ErbB4. Isoform-specific tissue distribution and differential processing in response to phorbol ester. The Journal of biological chemistry 272, 26761–26768 (1997). 9334263 10.1074/jbc.272.42.26761

[b36] Rio C., Buxbaum J. D., Peschon J. J. & Corfas G. Tumor necrosis factor-alpha-converting enzyme is required for cleavage of erbB4/HER4. The Journal of biological chemistry 275, 10379–10387 (2000). 10744726 10.1074/jbc.275.14.10379

[b37] Cheng Q. C., Tikhomirov O., Zhou W. & Carpenter G. Ectodomain cleavage of ErbB-4: characterization of the cleavage site and m80 fragment. The Journal of biological chemistry 278, 38421–38427 (2003). 12869563 10.1074/jbc.M302111200

[b38] Fujita E. . Oligo-astheno-teratozoospermia in mice lacking RA175/TSLC1/SynCAM/IGSF4A, a cell adhesion molecule in the immunoglobulin superfamily. Molecular and cellular biology 26, 718–726 (2006). 16382161 10.1128/MCB.26.2.718-726.2006 PMC1346906

[b39] van der Weyden L. . Loss of TSLC1 causes male infertility due to a defect at the spermatid stage of spermatogenesis. Molecular and cellular biology 26, 3595–3609 (2006). 16611999 10.1128/MCB.26.9.3595-3609.2006 PMC1447413

[b40] Kuramochi M. . TSLC1 is a tumor-suppressor gene in human non-small-cell lung cancer. Nature genetics 27, 427–430 (2001). 11279526 10.1038/86934

[b41] Zhang H., Li X. J., Martin D. B. & Aebersold R. Identification and quantification of N-linked glycoproteins using hydrazide chemistry, stable isotope labeling and mass spectrometry. Nature biotechnology 21, 660–666 (2003). 10.1038/nbt827 12754519

[b42] Nagano K., Shinkawa T., Kato K., Inomata N., Yabuki N. & Haramura M. Distinct cell surface proteome profiling by biotin labeling and glycoprotein capturing. Journal of proteomics 74, 1985–1993 (2011). 21621025 10.1016/j.jprot.2011.05.019

