# Supplementary material for: Mechanistic insights into ectodomain shedding: susceptibility of CADM1 adhesion molecule is determined by alternative splicing and O-glycosylation
Source: Sci Rep. 2017 Apr 10;7:46174. doi: 10.1038/srep46174 (PMC5385562; doi:10.1038/srep46174)
Supplement: Supplementary Information [file srep46174-s1.pdf]

Supplementary Information for

Mechanistic insights into ectodomain shedding: susceptibility of CADM1 adhesion molecule is determined by alternative splicing and *O*-glycosylation

Kyoko Shirakabe, Takuya Omura, Yoshio Shibagaki, Emiko Mihara,

Keiichi Homma, Yukinari Kato, Akihiko Yoshimura, Yoshinori Murakami,

Junichi Takagi, Seisuke Hattori, and Yoshihiro Ogawa

Supplementary Fig. S1

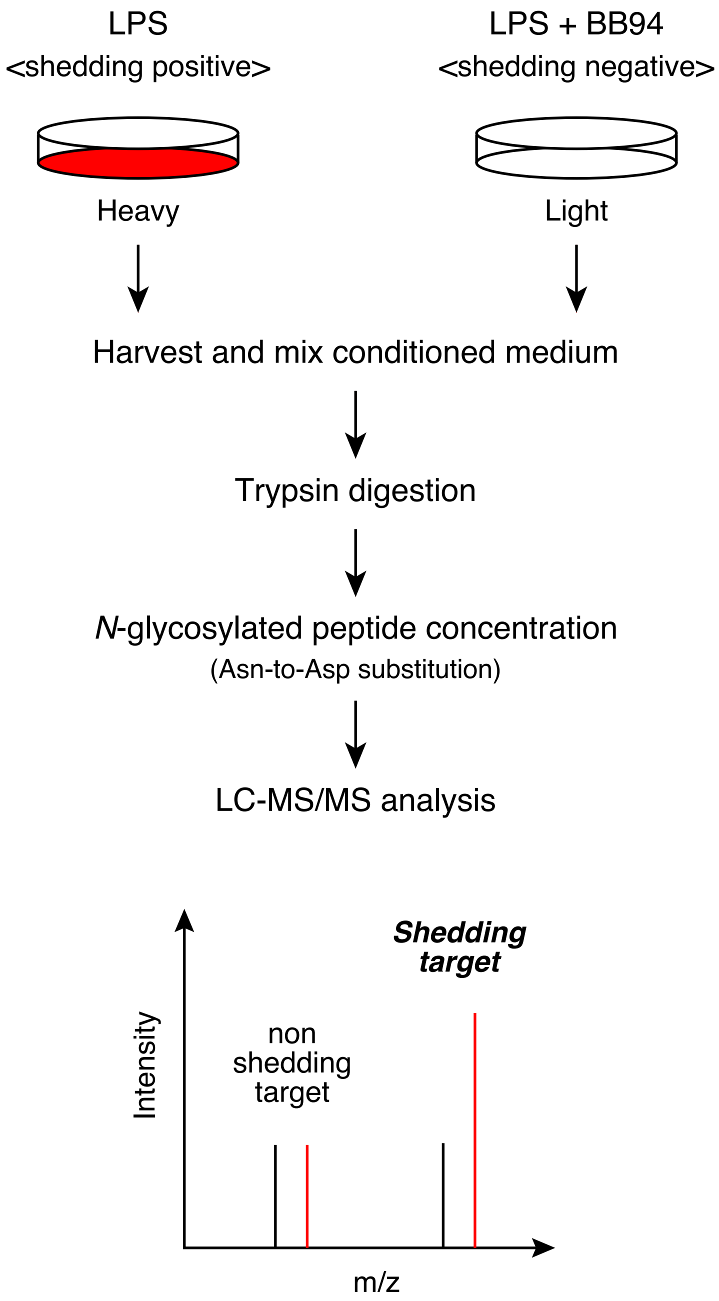

Supplementary Figure S1. Schematic diagram of proteomic screening of shedding targets in LPS-stimulated Raw 264.7 macrophage cells using stable isotope-based quantitative method called SILAC.

Supplementary Fig. S2

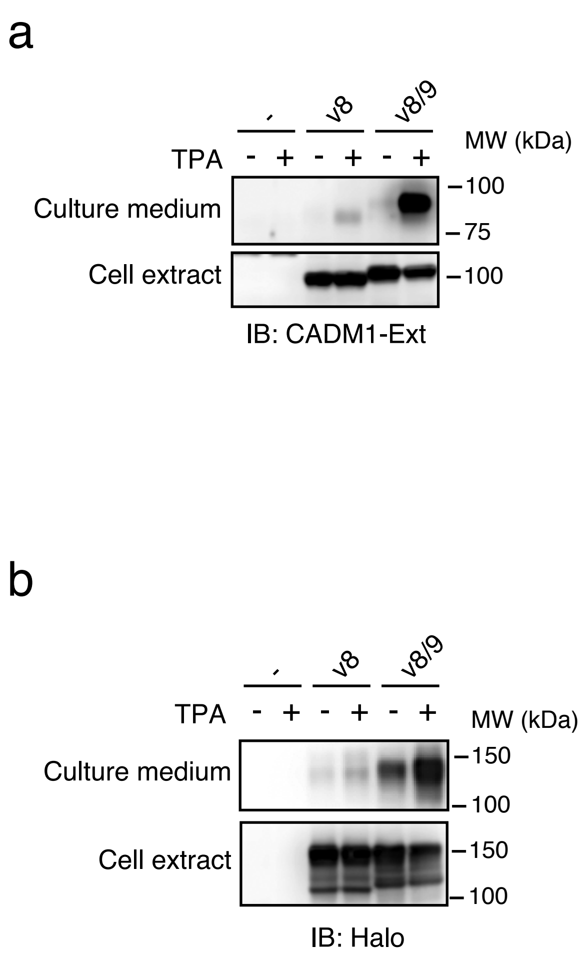

Supplementary Figure S2. Shedding susceptibility of CADM1 variants in different cell lines.
(a) Stable cell lines expressing v8 or v8/9 CADM1 in human small cell lung carcinoma SBC-5 cells were treated with (+) or without (-) 200 ng/ml of TPA, a potent and universal shedding inducer, for 60 min. Both cell extracts (Cell extract) and culture supernatants (Cuture medium) were subjected to Western blotting with an anti-CADM1 antibody. (b) MDCK cells expressing N-terminally Halo-tagged v8 or v8/9 CADM1 were treated with TPA for 60 min, and cell extracts and culture supernatants were subjected to Western blotting with an anti-Halo antibody.

Supplementary Fig. S3

Homo sapiens (Human)	DTTATTEPAVH
Mus musculus (Mouse)	DTTATTEPAVH
Rattus norvegicus (Rat)	DTTATTEPAVH
Canis lupus familiaris (Dog)	DTAATTEPAVH
Equus caballus (Horse)	DTTATTEPAVH
Monodelphis domestica (Opossum)	DTTATTEPAVH
Meleagris gallopavo (Turkey)	CVDTTATTEPAVH
Taeniopygia guttata (Zebra finch)	DTTATTEPAVH
Anolis carolinensis (Chameleon)	DTTAETEPAVH
Pelodiscus sinensis (Softshell turtle)	CVDTTATTEPAVH
Xiphophorus maculates (Southern platyfish)	DTVPSAEPAAH
Oreochromis niloticus (Nile tilapia)	ETAPSTEPAAH
Danio rerio (Zebrafish)	DAAPSTEAAAH

Supplementary Figure S3. Mouse exon 9 is evolutionarily conserved from fish to human. The amino acid sequences of CADM1 orthologs corresponding to mouse exon 9 are shown. All the sequences are encoded by single exons.

Supplementary Fig. S4

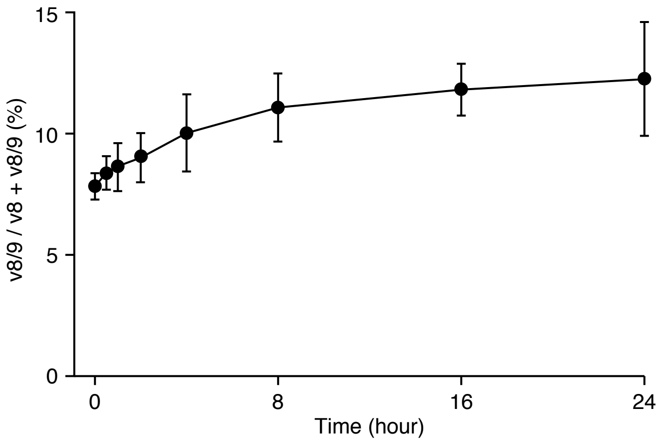

Supplementary Figure S4. The relative amount of v8/9 CADM1 mRNA in LPS-stimulated Raw 264.7 cells. Raw 264.7 cells were treated with LPS for up to 24 hours, and the amount of both v8 and v8/9 CADM1 mRNAs expressed in the cells were quantified. The relative abundances of v8/9 CADM1 mRNA were plotted. Each data point represents the mean of six independent experiments. The error bars indicate ± SD.

Supplementary Fig. S5

a

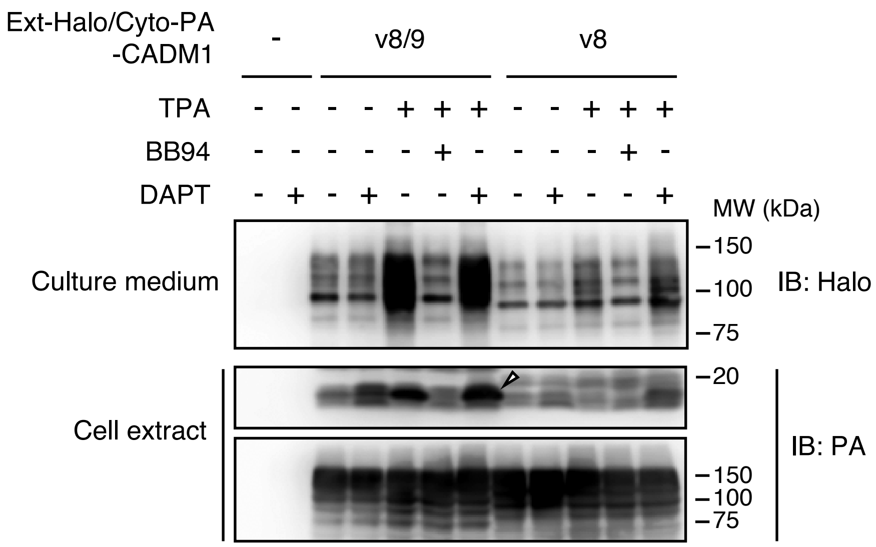

b

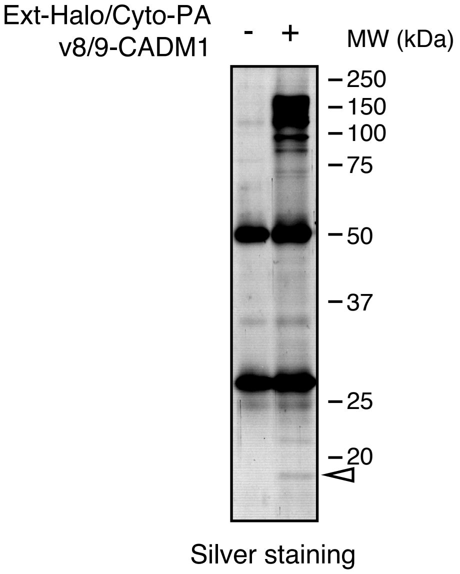

c

	Asp	Asn	Ser	Gln	Thr	Gly	Glu	His	Ala	Arg	Tyr	Pec	Pro	Met	Val	Trp	Phe	Ile	Lys	Leu
1	0.197	0.000	1.033	0.640	0.459	0.249	0.109	0.000	0.261	0.165	0.123	0.050	0.100	0.083	0.164	0.022	0.071	0.000	0.138	0.000
2	0.200	0.158	0.568	0.442	0.439	0.214	0.093	0.000	0.244	0.183	0.121	0.068	0.129	0.023	0.230	0.000	0.104	0.107	0.154	0.163
3	0.070	0.043	0.000	0.438	0.499	0.262	0.107	0.000	0.589	0.188	0.124	0.058	0.110	0.000	0.188	0.024	0.092	0.269	0.125	0.152
4	0.080	0.046	0.382	0.422	0.443	0.399	0.159	0.055	0.453	0.165	0.135	0.035	0.172	0.048	0.158	0.000	0.125	0.302	0.151	0.207
5	0.158	0.147	0.350	0.431	0.440	0.361	0.309	0.000	0.378	0.000	0.146	0.024	0.132	0.057	0.230	0.000	0.132	0.328	0.189	0.261
6	0.090	0.000	0.000	0.413	0.484	0.382	0.426	0.023	0.378	0.149	0.170	0.054	0.151	0.038	0.230	0.000	0.144	0.294	0.175	0.266
7	0.096	0.087	0.397	0.445	0.557	0.417	0.374	0.000	0.362	0.135	0.152	0.051	0.132	0.026	0.274	0.039	0.154	0.288	0.254	0.315
8	0.085	0.090	0.458	0.442	0.650	0.427	0.281	0.000	0.381	0.157	0.156	0.049	0.127	0.040	0.292	0.000	0.131	0.346	0.134	0.222
9	0.114	0.063	0.458	0.440	0.641	0.430	0.247	0.000	0.438	0.152	0.171	0.000	0.175	0.039	0.320	0.000	0.178	0.605	0.232	0.311
10	0.103	0.066	0.000	0.394	0.526	0.497	0.239	0.000	0.433	0.157	0.182	0.000	0.153	0.023	0.308	0.000	0.177	0.609	0.214	0.346
11	0.129	0.063	0.468	0.427	0.450	0.490	0.251	0.021	0.581	0.159	0.178	0.024	0.137	0.039	0.312	0.025	0.209	0.518	0.248	0.334
12	0.124	0.063	0.509	0.432	0.503	0.490	0.244	0.000	0.612	0.168	0.208	0.039	0.151	0.000	0.487	0.000	0.219	0.442	0.239	0.360

Supplementary Figure S5. Determination of the shedding cleavage site of v8/9 CADM1. (a) The N-terminal Halo-tagged and cytoplasmic PA-tagged v8 or v8/9 CADM1 was expressed in HEK 293 cells, and cells were treated with TPA, BB94, and/or DAPT (an intramembrane proteolysis inhibitor) for 90 min as indicated. Both cell extracts and culture supernatants were subjected to Western blotting with an anti-Halo antibody or an anti-PA antibody. White triangle indicates the accumulated ~15 kDa membrane-remaining shedding product. (b) ~15 kDa shedding product was affinity purified from the extract of HEK 293 cells expressing cytoplasmic PA-tagged v8/9 CADM1, separated by SDS-PAGE, and visualized by silver staining. White triangle indicates the ~15 kDa shedding product. (c) The N-terminal sequence analyses of the ~15 kDa shedding product. The table shows the recovery of PTH amino acids (in pmol) in each cycle. The amino acids identified by the peak analysis program are shaded.

Supplementary Fig. S6

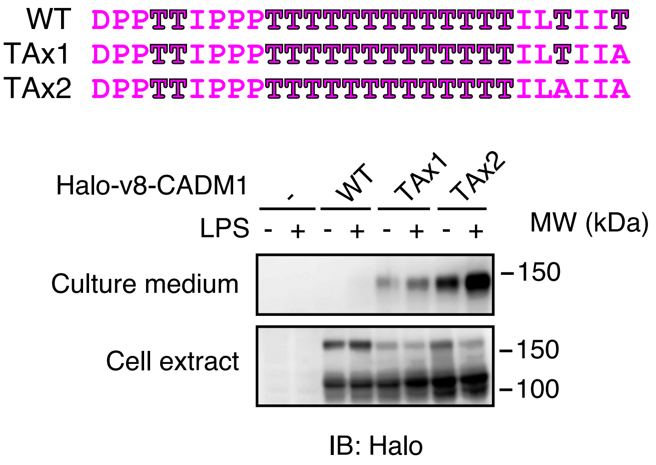

Supplementary Figure S6. Shedding susceptibility of alanine substitution mutants of v8 CADM1. Raw 264.7 cells expressing N-terminally Halo-tagged v8 CADM1 mutants were treated with LPS for 60 min, and cell extracts and culture supernatants were subjected to Western blotting with an anti-Halo antibody. The amino acid sequences of substitution mutants are indicated above.

Methods

Cell lines and chemicals.

Stable cell lines expressing v8 or v8/9 CADM1 in human small cell lung carcinoma SBC-5 cells were established previously [1]. These stable cell lines, canine MDCK epithelial cells, and human embryonic kidney 293 cells were cultured in DMEM supplemented with 10% fetal bovine serum and antibiotics. TPA (12-*O*-Tetradecanoylphorbol 13-acetate) was purchased from Merck Millipore (Darmstadt, Germany). DAPT was purchased from Sigma-Aldrich.

Analysis of CADM1 orthologs.

CADM1 orthologs listed in the OrthoDB version 8 [2] were aligned by the MUSCLE program version 3.5 [3].

Quantification of CADM1 variant mRNAs.

cDNA fragments of CADM1 corresponding to exons 7-11 were amplified by PCR using primers described previously [1] from cDNA libraries of Raw 264.7 cells treated with LPS for 0.5-24 hours. The PCR products were separated and quantified using 2100 bioanalyzer (Agilent, Santa Clara, CA).

N-terminal sequencing of a membrane-remaining shedding product of CADM1.

HEK 293 cells expressing cytoplasmic PA-tagged v8/9-CADM1 were treated with 200 ng/ml TPA, 10 μM BB94, and 10 μM DAPT for 90 min and extracted.

The extract was incubated with anti-PA tag antibody beads (WAKO) at 4˚C overnight, washed by Tris-buffered saline, and eluted by boiling in the saline containing 2% SDS and 40 mM DTT. Eluted proteins were separated by SDS-PAGE, blotted onto PVDF membrane, and stained with Coomassie Blue. ~15 kDa membrane-remaining shedding product of CADM1 was excised and subjected to automated Edman degradation on a protein sequencer Procise 491 cLC (Thermo Fisher Scientific).

References

1. Kikuchi S, *et al*. Expression of a splicing variant of the CADM1 specific to small cell lung cancer. *Cancer science* **103**, 1051-1057 (2012).

2. Kriventseva EV, *et al*. OrthoDB v8: update of the hierarchical catalog of orthologs and the underlying free software. *Nucleic acids research* **43**, D250-256 (2015).

3. Edgar RC. MUSCLE: multiple sequence alignment with high accuracy and high throughput. *Nucleic acids research* **32**, 1792-1797 (2004).
